# Cervical Epidural and Subarachnoid Catheter Placement in Standing Adult Horses

**DOI:** 10.3389/fvets.2020.00232

**Published:** 2020-05-14

**Authors:** Samuel D. Hurcombe, Tate B. Morris, Ashley R. VanderBroek, Perry Habecker, Kathryn Wulster, Klaus Hopster

**Affiliations:** Department of Clinical Sciences, New Bolton Center, University of Pennsylvania, Philadelphia, PA, United States

**Keywords:** cervical epidural, subarachnoid space, ultrasound-guided, cerebrospinal fluid, catheter, horse

## Abstract

Horses underwent either cervical epidural space (CES) catheterization or subarachnoid space (SAS) catheterization while restrained in stocks, under deep sedation (detomidine and morphine) and local anesthesia (mepivacaine 2%) block. Catheters were placed under ultrasound guidance with visualization of the dura, SAS, and spinal cord between the first (C1) and second (C2) cervical vertebrae. Following sedation and sterile skin preparation, operator 1 placed under ultrasound guidance, a 6- or 8-inch Tuohy needle with the bevel oriented caudally. For CES, a 6-inch Touhy needle was used with the hanging drop technique to detect negative pressure, and operator 2 then passed the epidural catheter into the CES. For SAS, following puncture of the dura, cerebrospinal fluid (CSF) was aspirated prior to placement of the epidural catheter. Placement into either CES or SAS was confirmed with plain and contrast radiography. Catheters were wrapped for the duration of the study. CSF cytology was assessed up to every 24 h for the study period. Horses were assessed daily for signs of discomfort, neck pain, catheter insertion site swelling, or changes in behavior. A complete postmortem assessment of the spinal tissues was performed at the end of the study period (72 h). Two horses had CES catheters and five horses had SAS catheters placed successfully. All horses tolerated the catheter well for the duration of the study with no signs of discomfort. Ultrasound was essential to assist placement, and radiography confirmed the anatomical location of the catheters. CSF parameters did not change over the study period (*P* > 0.9). There was evidence of mild meningeal acute inflammation in one horse and hemorrhage in another consistent with mechanical trauma. Placement of an indwelling CES or SAS catheter appears to be safe, technically simple, and well tolerated in standing sedated normal horses.

## Introduction

Disorders of the cervical spine are commonly reported in people with neck pain, cervical radiculopathy, and discomfort, necessitating therapeutic intervention (1). Cervical spine analgesia provision using either an indwelling epidural catheter or local injection is commonly performed using imaging guidance such as epidurography (2) or ultrasound in people with cervical spine disorders including intervertebral disc disease and foraminal or spinal stenosis (3). Recognition of neck disease and pain in horses is increasingly being investigated as causes of poor performance, postural changes, lameness, behavioral changes, and spinal ataxia (4, 5). While caudal epidural catheterization and analgesia provision can be used for painful orthopedic and urogenital disorders in horses (6), painful disorders of the forelimbs such as laminitis and other orthopedic conditions are often challenging to treat with spinal analgesia that specifically targets spinal cord segments and peripheral nerves of the brachial intumescence in horses. There is a heavy reliance on systemic therapies over spinal analgesia that all have potential adverse effects.

Currently, in standing horses, cervical spine radiographs, cerebrospinal fluid (CSF) assessment, and rarely standing myelography (7, 8) are diagnostics available to veterinarians to evaluate the cause of cervical spine disease. Experimentally, epiduroscopy and myeloscopy have also been described (9, 10). A technique for CSF collection using ultrasound guidance from between the first and second cervical vertebrae (C1–2) has been described and adopted into clinical practice (11). The advantage of this technique over atlanto-occipital space collection is there is no requirement for general anesthesia and having the head in flexion. The advantage of standing C1–2 aspiration over lumbosacral CSF collection is that the clinician can visualize the dura, subarachnoid space (SAS), and spinal cord as well as needle placement, and there is a shorter distance of needle passage to enter the SAS. Further, there is a perceived increased safety to personnel involved as often lumbosacral puncture can result in violent reactions upon puncture of the dura (12, 13) which appear to be less so in the cervical region for reasons unknown (11, 14).

Because visualization of the dura, SAS, and spinal cord at C1–2 using ultrasound is simple, we hypothesized that placement of an indwelling catheter to either the epidural space or SAS would be achievable in the standing horse. Further, that if catheterization was well-tolerated and safe, it may offer an avenue for therapeutic administration of targeted spinal analgesia for painful conditions of the neck and forelimbs in future studies as well as advanced imaging in the standing horse.

The goal of this study was to evaluate the feasibility of placing a long-term epidural catheter into the cervical epidural space (CES) or SAS in standing horses. Our objectives were to determine with imaging and pathology the exact location of catheter tip, whether placement for 72 h would result in clinical, clinicopathological, and gross pathological evidence of meningitis or CSF leakage, and to see how the implant would be tolerated by the horse.

## Materials and Methods

### Study Design

#### Prospective, Descriptive Study

Adult horses (*n* = 7), <1 year of age, were enrolled in this study. After obtaining owners' consent, horses were donated to New Bolton Center at the University of Pennsylvania for research following diagnosis of chronic, incurable, disease (five chronic unresolved lameness, one upper airway nasal passage disorder, one chronic recurrent cellulitis). This study was approved by the institutional animal care and use committee (protocol #805949) and adheres to the principles for the humane treatment of animals as stated by the National Institutes of Health guidelines.

### Procedure(s)

#### Patient Preparation

Horses were restrained in standing stocks, and an experienced handler maintained control of the head. Intravenous flunixin-meglumine [1.1 mg/kg intravenous (IV)] and detomidine sedation were administered (0.01 mg/kg, IV). The mane was braided and secured with rubber bands to allow exposure of the proximal cervical neck region.

With horses restrained in stocks, a handler maintained control of the head using a halter and held in a neutral head up position, trying to avoid excessive flexion or extension. An ultrasound of the proximal cervical neck was used to identify the space between the first cervical vertebra (C1) and the second cervical vertebra (C2) according to Pease et al. (11). Once identified, a 2-inch by 2-inch area was clipped and aseptically prepared using chlorhexidine gluconate (4%) for 5 min, followed by isopropyl alcohol (70%).

Mepivicaine HCl (2%) was then aseptically drawn into a 10-ml syringe and a 22-gauge, 1.5-inch needle was used to infiltrate the proposed area of spinal needle insertion fanning in a proximal to distal direction.

A second surgical scrub with chlorhexidine gluconate was then performed for 3 min followed by isopropyl alcohol. When horses were judged to be adequately sedated by showing minimal response to tactile or auditory stimuli, morphine sulfate (0.1 mg/kg, IV single bolus) was administered.

The ultrasound probe (Philips Lumify, C5-2 broadband curved array 2–5 MHz probe) was then dressed in a sterile plastic sleeve and coupling gel.

A single operator (SDH) then held both probe and a 17-gauge, 6-inch (epidural) or 8-inch (subarachnoid) curve-tipped spinal needle with stylet (Tuohy).

#### Catheter Placement Technique

With the horses' head held in a neutral position with axial alignment, the ultrasound probe was oriented in a vertical (dorsal to ventral) direction to visualize the spinal cord, SAS, and dura between C1 and C2 on the proximolateral neck.

For SAS placement, an 8-inch Tuohy needle was advanced 10–15 degrees rostrocaudal off perpendicular to the neck until the needle tip was observed to rest adjacent the dura on ultrasound. The probe was then set down and the stylet removed. A 6-ml slip tip syringe was attached to the needle by a second operator (KH), and while maintaining slight negative pressure, the needle operator advanced the needle to enter the SAS with the curved bevel facing caudally. Successful placement into the SAS was confirmed by freely aspirating CSF.

While holding the Tuohy needle in place, the second operator (KH) then began to advance an epidural catheter (FlexBlock^TM^, 19-gauge, 60 cm, Arrow International Inc., Reading, PA, USA). Generally, the catheter was advanced beyond the first point of resistance which was approximately as the catheter exited the needle tip as estimated by markings along both the Tuohy needle and epidural catheter. If a second point of resistance was encountered, the catheter was not advanced any further.

For CES placement, a 6-inch Tuohy needle was advanced perpendicular to the neck in a slight (10–15 degrees) rostrocaudal direction with the bevel facing caudally until the needle tip was ~5 mm from the dura on ultrasound. The probe was set down, the stylet was removed, and a hanging drop technique employed using sterile 0.9% sodium chloride to detect negative pressure upon entering the epidural space. While holding the Tuohy needle in place, the second operator advanced an epidural catheter until a second resistance was encountered.

In both SAS or CES placement, the Tuohy needle was then withdrawn over the remaining length of the catheter, and the indwelling catheter was secured with plastic eyed butterflies and sutured in the skin using 2–0 Prolene. A microfilter and sterile injection cap were attached to the catheter and placed into the adhesive catheter hub which was placed caudal to the insertion site on the neck. Skin glue was placed at the insertion site of the catheter, and sterile gauze swabs were then placed over the insertion site, and a neck bandage was placed using Elastikon.

#### Imaging

After SAS or CES catheter placement, plain radiography views of the head and cranial cervical spine were obtained. Lateral projections with the plate on the side of insertion were performed. Catheter placement within the spinal canal and catheter tip location were documented. Next, 5 ml non-ionic contrast agent (Iohexol® 300 mg/ml) was injected into the catheter over 1 min, and additional radiographs were used to confirm placement within the CES or SAS.

Horses were then restrained for 20 min in standing stocks with the head and neck elevated to a neutral position until the effects of sedation were no longer evident.

Horses returned to a box stall for monitoring, including signs of discomfort, neck pain/stiffness, reduced range of motion, ataxia, changes in neurologic status, and colic. Horses were fed free choice grass hay on the ground and provided water for the following 72 h. Catheter insertion sites were visually inspected twice daily for signs of heat, pain, and inflammation. SAS catheters were flushed with 2 ml 0.9% NaCl once a day (catheter volume 0.35 ml) to help maintain patency.

#### Cerebrospinal Fluid Assessment

For horses with SAS catheters, CSF was collected from the catheter for cytological assessment at placement then up to every 24 h for the duration of the study (catheter placement = 0 h, 24, 48, and/or 72 h). One milliliter of CSF was aspirated and discarded, then 2–4 ml of CSF was collected and placed into ethylenediaminetetraacetic acid (EDTA) tubes for total nucleated cell count, cell differential, and protein concentration.

#### Pathology

Following CT imaging, horses were humanely euthanized using xylazine HCl (1 mg/kg IV) for sedation, ketamine HCl (2 mg/kg IV) for anesthetic induction, then potassium chloride (1.5 mmol/kg IV bolus) for euthanasia as accepted by the American Veterinary Medical Association. The catheter insertion site and cervical spinal cord were examined grossly and histologically for evidence of hemorrhage, suppuration, abscessation, and inflammation.

### Statistical Methods

All data were entered into a spreadsheet and analyzed using commercially available software (Microsoft Excel, Redmond, WA, and GraphPad Prism v8, San Diego, CA, USA). Continuous data were assessed for normality using the Shapiro–Wilk Statistic. Changes in CSF cytological parameters were assessed using a nonparametric one-way ANOVA for repeated measures (Friedman test). Significance was set at *P* < 0.05.

## Results

Five horses had SAS catheters placed and two horses had CES catheters placed. All but one horse was successfully catheterized using local anesthesia and sedative analgesia while standing. A single horse was too reactive to SAS catheter placement despite deep sedation and then became too ataxic related to heavy sedation for safe placement. This horse was subsequently anesthetized with ketamine HCl (2.2 mg/kg, IV), and the catheter was placed in the left lateral recumbency. This horse recovered uneventful from anesthesia after 35 min.

In all horses, the dura, SAS, and spinal cord were easily identified between C1 and C2 using ultrasonography as described by Pease et al. (11). In all horses, the needle trajectory was visualized such that the needle point was placed in the desired location either 3–5 mm from the dura or observed to minimally deform the dura. Using an 8-inch Tuohy needle had sufficient length to allow the operator to attempt rostrocaudal (15 degrees) insertion rather than true perpendicular insertion to help reduce the angle of catheter passage out of the needle bevel. In horse 1, the 6-inch needle did not allow for sufficient angulation and penetration into the SAS, resulting in CES placement.

For SAS catheter placement, 2/5 (40%) reacted adversely to dural puncture characterized by lifting the head and neck, and in both horses, the needle was withdrawn immediately to avoid inadvertent trauma to the spinal cord. Following additional sedatives, the needle was replaced under ultrasound guidance, and both horses tolerated a second dural puncture. The remaining 3/5 (60%) horses did not react to dural puncture and CSF aspiration.

For CES catheter placement, both horses did not react to needle placement into the epidural space. The hanging drop technique was useful in horse 7 in identifying needle tip placement into the CES.

**Figure 1:**
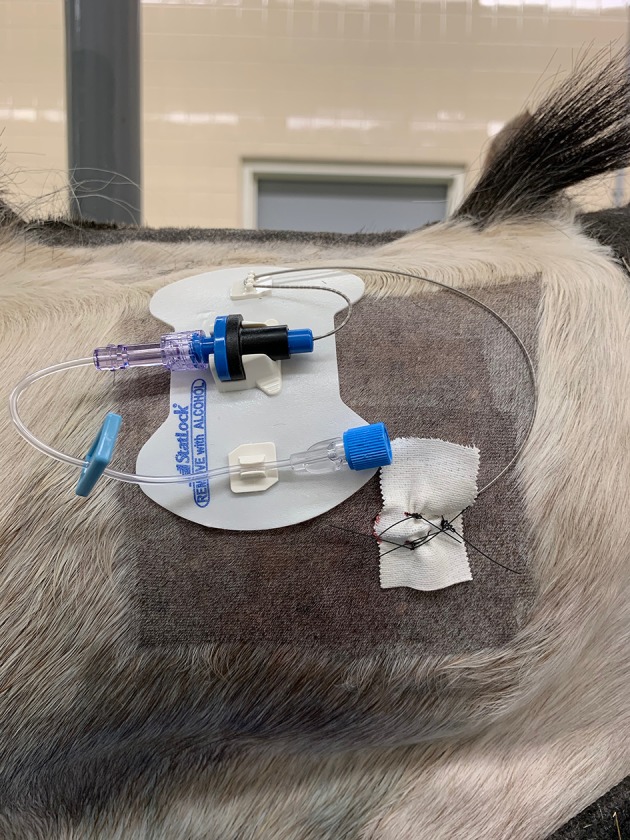
Photo showing the position of the catheter on the dorsolateral neck region fixed in place within a self-adhesive hub to the skin. The catheter insertion site is fixed to the horse using tape and three skin sutures. The head is to the right of the image (Horse 5).

In horses with SAS catheters, 4/5 (80%) horses reacted to catheter insertion and passage within the SAS. Horses typically twitched the head and neck as the catheter was inserted. In 3/5 horses (horses 2, 4, and 6), resistance to catheter passage was encountered at two locations, once as the catheter exited the needle tip and again approximately after 25% of the catheter length was passed. When a second point of resistance was encountered, passage was stopped, and the catheter was fixed in place. Estimation of catheter insertion lengths was made based on markings along the catheter. In 1/5 horses (horse 5), the second point of resistance occurred after 50% (30 cm) of the catheter length had advanced, and in one other horse (horse 3), the second point of resistance occurred after 75% (45 cm) of the catheter length was passed. Catheters were not trimmed, and any excess length was looped into the adhesive pad attached to the horses' neck (Figure 1).

Following placement of the catheters, each catheter was attached to an injection port and self-adherent pad to the side of the neck of insertion (Figure 1). Sterile gauze swabs and an adhesive dressing were placed to cover the catheter insertion and injection ports. The median time taken to place the catheter (SAS or CES) following sterile preparation and block of the skin and musculature was 14 min.

### Radiographic Assessment

**Figure 2:**
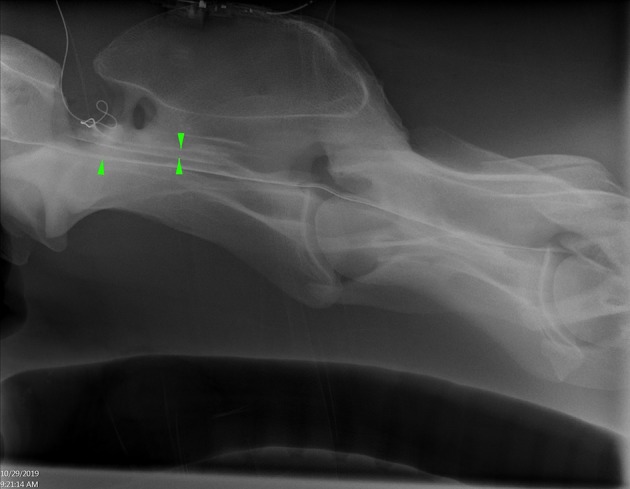
Lateral radiograph of caudal C1 to cranial C4 showing radiopaque catheter at the level of C1–2. Positive contrast outlines the ventral border of the spinal canal (single arrowhead) and a faint outline of the spinal cord (opposing arrow heads) (Horse 6).

**Figure 3:**
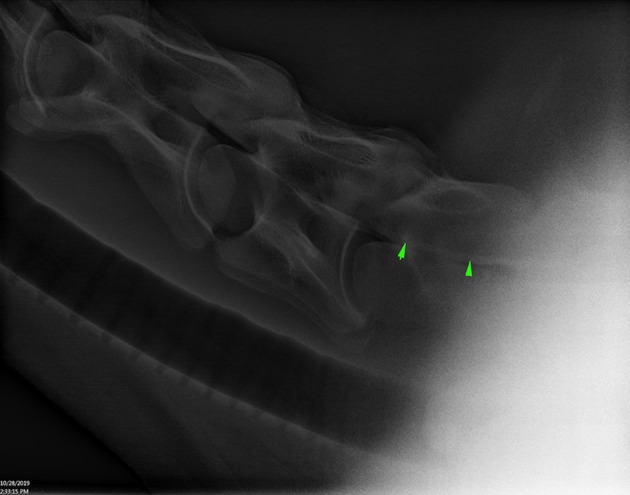
Lateral radiograph of caudal C4 to cranial T1 showing positive contrast pooling along the ventral spinal canal of C7 (arrow heads) (Horse 6).

**Figure 4:**
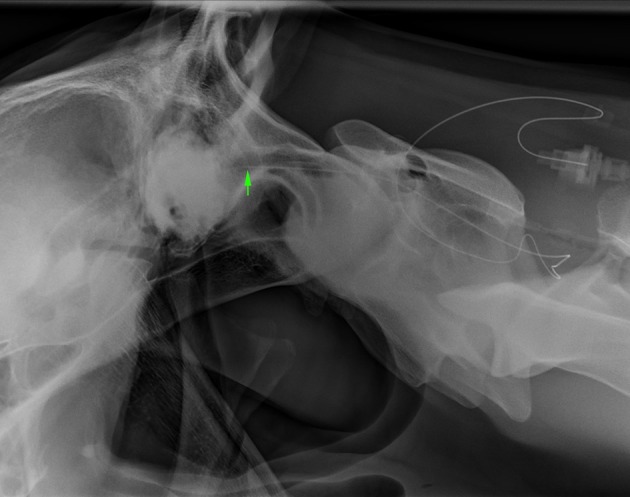
Lateral radiograph of caudal skull to cranial C2. The radiopaque catheter is seen running rostrally from the insertion at C1–2 with the catheter tip in the rostrodorsal occipit area (arrowhead) (Horse 5).

**Figure 5:**
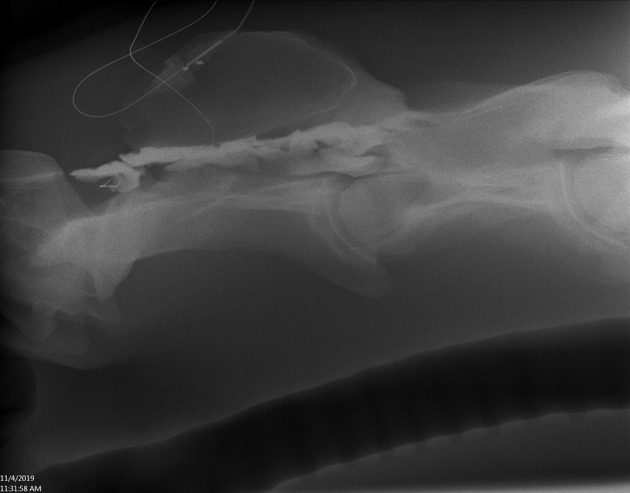
Lateral radiograph of caudal C1 to cranial C4. Positive contrast outlines the epidural/subdural space of C2 (Horse 7).

All horses received plain radiographic imaging of the cervical spine to assess the location of the catheter tip within the spinal canal before and after positive contrast administration. Table 1 describes the location based on cervical vertebrae for all horses included. Following survey radiographs, 5 ml sterile iohexol (300 mg/ml) was injected into the catheter and the radiographs were repeated to confirm the anatomical location of the catheter tip (epidural, subdural, or subarachnoid) based on previously published criteria for contrast accumulation (15, 16). Horse 1 was confirmed to be epidural despite attempting subarachnoid placement. This was the first horse, and it was determined that the 15-cm (6-inch) Tuohy needle was too short to penetrate the dura despite seeing the needle reaching the dura mater on ultrasound. For horses 2–6, a 20-cm (8-inch) Tuohy needle aided in correct entry into the SAS and confirmed by aspiration of CSF as well as contrast radiography highlighting a faint outline of the cervical spinal cord (Figures 2, 3). In Horse 5, the catheter tip had advanced rostrally to the rostral dorsal occipit (Figure 4). No contrast was given to this horse. Horse 7 had a CES catheter placed intentionally using the hanging drop technique and confirmed by contrast radiography (Figure 5).

**Table 1:** Demographic information, location of catheter placement, placement details, and radiographic location of catheter tip in horses undergoing subarachnoid or cervical epidural catheter placement.

**Horse number**	**Signalment**	**Horse position for catheter placement**	**Location of catheter placement**	**Number of attempts (needle placement)**	**Time taken for catheter placement (after sterile preparation)**	**Radiographic location of catheter tip**
1	2-year, Standardbred, colt	Standing	CES	4	22	Dorsal mid C2 and double back on itself. Contrast confirmed CES placement.
2	10-year, Thoroughbred, mare	Standing	SAS	3	16	Dorsal, caudal C2. Contrast confirmed SAS placement.
3	12-year, Thoroughbred, mare	Standing	SAS	2	12	Dorsal cranial C4. Contrast confirmed SAS placement.
4	8-year, Thoroughbred, gelding	Left lateral recumbency	SAS	3 (2 standing; 1 under general anesthesia)	18	Dorsal caudal C2. Contrast confirmed SAS placement.
5	5-year, Thoroughbred, gelding	Standing	SAS	2	12	Dorsal caudal occiput. No contrast given.
6	4-year, Thoroughbred, gelding	Standing	SAS	1	14	Dorsal mid C2. Contrast confirmed SAS placement.
7	4-year, Thoroughbred, gelding	Standing	CES	1	9	Dorsal mid C2. Contrast confirmed CES or subdural placement.

*SAS, subarachnoid space; CES, cervical epidural space; C2, second cervical vertebra; C3, third cervical vertebra; C4, fourth cervical vertebra*.

All horses subjectively tolerated the indwelling catheter very well. While specific pain scores were not recorded in these horses, their behavior, level of interaction with people, apparent comfort eating hay from the ground, and willingness to ambulate in the stall were consistent and deemed appropriate among all horses. When catheter insertion sites were visually assessed, subjective range of motion of the neck was also performed without any evidence of neck discomfort. Objective assessment of heart rate, temperature, and respiratory rate monitored every 8–12 h remained within normal limits for all horses. Horses were housed for 3 days with indwelling catheters prior to humane euthanasia and pathology assessment.

For horses with SAS catheters, CSF analysis was unremarkable for the duration of the study (Table 2) with mean nucleated cell counts <5 cells/μl and protein <100 mg/dl consistently over the 3-day experimental period.

**Table 2:** Cerebrospinal fluid cytological findings in horses with indwelling subarachnoid space catheters (*n* = 5).

**Horse number**	**Total nucleated cell count (/μl) Placement**	**Total nucleated cell count (/μl) 24 h**	**Total nucleated cell count (/μl) 48 h**	**Total nucleated cell count (/μl) 72 h**	***P* value**
2	6	–	–	4	–
3	1	2	0	1	–
4	7	4	5	5	–
5	3	2	–	5	–
6	6	4	–	0	–
Mean (n)	4.6 (5)	3 (4)	2.5 (2)	3 (5)	0.92^*^
**Horse number**	**CSF protein (mg/dl) Placement**	**CSF protein (mg/dl) 24 h**	**CSF protein (mg/dl) 48 h**	**CSF protein (mg/dl) 72 h**	***P*** **value**
2	60	–	–	40	–
3	40	50	50	70	–
4	20	20	10	10	–
5	50	40	–	40	–
6	80	10	–	8	–
Mean (*n*)	50 (5)	30 (4)	30 (2)	34 (5)	>0.99^*^

* *Not significant (P > 0.05)*.

### Pathology

**Figure 6:**
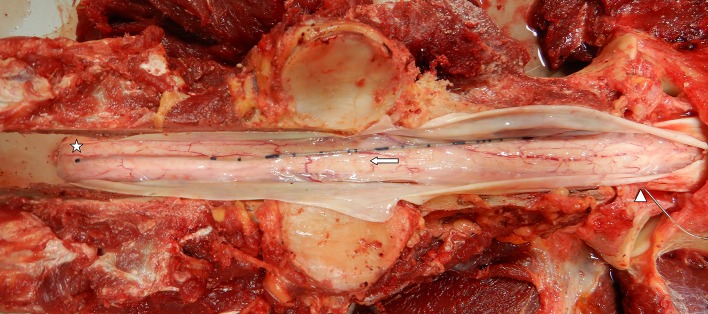
Gross prosection specimen of a subarachnoid catheter dural insertion at C1–2 (arrowhead), passage along the subarachnoid space (arrow), and catheter tip located along the dorsal spinal cord at the level of cranial C4 (star). Note the dura mater has been incised and reflected to show the catheter (Horse 3).

In most horses, minor resolving hemorrhage was observed in the subcutis and cervical musculature associated with mepivacaine infiltration and/or Touhy needle placement. Each horse had a detailed examination to identify the location of the catheter tip. Figure 6 is an example of *in situ* assessment within the spinal canal. In this case (horse 3), the catheter tip is located adjacent the spinal cord in the SAS at the level of C4. In horse 1, there was evidence of mild epidural inflammation and hemorrhage, consistent with mechanical trauma. This horse coincidently was also the case where four attempts at placement occurred. In horse 3, mild neutrophilic inflammation was found associated with the dura mater, arachnoid membrane, and perineurium of the C2 spinal cord rootlet at the level of dural puncture site. While not clinically apparent, this horse also had a rising CSF protein concentration at 72 h, although still below reference range (<100 mg/dl). There was no evidence of CSF leakage into the epidural or extra-axial spinal tissues.

## Discussion

Results of this technique study show that both CES and SAS catheter placement are feasible, repeatable, and well-tolerated in standing sedated horses. Ideally, three people are required to facilitate placement: an animal handler, ultrasound-guided needle placement operator, and catheter advancement operator. The authors emphasize the need for balanced neuroleptanalgesia and local anesthesia infiltration for successful placement of catheter, especially those in the SAS. Further, the authors encourage others to practice both acquiring the ultrasound image of the spinal cord at C1–2 and catheter placement technique on cadaver specimens to improve proficiency and minimize trauma in live animals.

Use of ultrasound was essential to identify the correct location for needle placement. As described previously (11), access to the subarachnoid and epidural space between C1 and C2 is easily observed. Ultrasound has also been shown to improve delivery of spinal analgesia/anesthesia in humans (17, 18) where imaging helps with both reducing the number of attempts to access the SAS and time of the procedure and patient comfort (19). Further, ultrasound has been shown to minimize repeated trauma and CSF contamination in horses when used for lumbosacral CSF collection, highlighting its utility in such procedures (20).

Some horses in this study undergoing SAS catheterization did react during the procedure, whereas neither horse with CES-placed catheters did. This is likely related to dural puncture and nociception stimulation, a well-recognized phenomenon in horses undergoing lumbosacral CSF aspiration (13). These reactions were transient, inconsistent, and managed with further sedation. While horses appeared to be normal following catheterization, post dural puncture headache (PDPH) is a well-described phenomenon in humans (21) related to leakage of CSF into the epidural space. Blood patch placement and use of cyclooxygenase 3 inhibitors (acetaminophen/paracetamol) are often successful in treating humans with PDPH (22); moreover, in humans, reported side effects of interlaminar or transforaminal epidural injection can include hypotension, neural injury, hemorrhage, infection, and infarction (23). Horses in this study received both morphine (along with detomidine) and a single periprocedural dose of flunixin meglumine which may have relieved any potential discomfort associated with the procedure.

Imaging with plain and contrast radiography was useful to identify epidural placement of catheters. For SAS placement, aspiration of CSF from the catheter confirms placement; however, small volumes of contrast were useful to identify intrathecal placement and location of the catheter tip. The authors recommend the use of contrast radiography for confirmation of CES placement (cervical epidurography). If CSF is aspirated from the catheter, SAS or subdural placement has likely occurred, confirmed with contrast radiography, or computed tomography.

Evaluation of the cervical spinal cord in standing horses using contrast with either robotic CT or plain myelography is another potential benefit of SAS catheterization. We showed that contrast can be administered in standing sedated horses and two-dimensional imaging performed. Further study in horses with clinical evidence of spinal ataxia would be needed to assess the diagnostic utility in documenting cord compression and other disorders.

The location of SAS catheter tip varied in the horses in this study. While the majority of catheters advanced to mid-caudal C2, in one horse, the catheter easily advanced to C4, and in another, there was rostral advancement to the level of the occipit. We speculate that the flexible catheter tip was redirected by an intrathecal structure (arachnoid fiber etc.) during advancement. The authors advocate imaging prior to contrast injection to confirm the location of the catheter tip as illustrated by horse 5. CSF was easily obtained from all SAS catheters regardless of location, and catheter tip location did not appear to cause any adverse responses in horses.

We propose that cervical epidural catheterization or intrathecal catheterization might offer a hospital-based option for spinal analgesia and anti-inflammatory therapy delivery to the neck and forelimbs but is yet to be determined. While the prevalence of neck pain or spinal ataxia secondary to advanced cervical facet osteoarthritis is unknown in horses, in humans, more than 33% of back pain patients are classified as neuropathic (24) necessitating analgesia. Tunneled epidural catheters have been placed for long-term use in people rehabilitating from cancer or in patients with prolonged pain following thoracic surgery (25).

In horses, further study is needed to determine the optimal injectate volume to reach clinically relevant anatomy (typically lower cervical region) for the treatment of lower neck and forelimb disorders as is described in people (26).

Other uses for SAS catheterization may include ease of repeated CSF sampling while minimizing/negating blood contamination from repeated puncture. A prior study in Gottingen minipigs showed the value of indwelling SAS catheter for studying the pharmacokinetics of drugs and their penetration into the central nervous system and CSF. In that study, catheters were left indwelling for 48 h (27). We showed that catheters placed for 72 h were well-tolerated, CSF was easily obtained in the conscious, unsedated animal, and incited minimal changes to CSF cytological parameters. The authors emphasize the importance of strict aseptic technique when placing catheters and speculate that catheters may be tolerated longer as is seen in other species but yet to be determined (28, 29).

In conclusion, placement of indwelling CES or SAS catheters appears to be safe, well-tolerated, and technically feasible in standing sedated adult horses. Future studies are needed to determine the value of long-term catheterization on spinal imaging or spinal analgesia provision in standing horses.

## Data Availability Statement

All medical records and research compliance records are available. Direct all requests to SH, hurcombe@vet.upenn.edu.

## Ethics Statement

The animal study was reviewed and approved by University of Pennsylvania IACUC protocol number #805949.

## Author Contributions

SH and KH contributed to concept design, performed all procedures, and collected all data. TM and AV assisted in data collection. PH performed all postmortem examinations. KW provided imaging consultation and interpretation. SH performed statistical analysis and preparation of the manuscript. KH, TM, and AV provided further manuscript revision. All authors contributed to manuscript revision and read and approved the submitted version.

## Conflict of Interest

All authors are affiliated with the same institution as Recent Advancement in Equine Anesthesia topic editors BH and KH: School of Veterinary Medicine, University of Pennsylvania, Philadelphia, PA, United States. KH is a coauthor and topic editor.
